# Bioimpedance spectroscopy for fluid status assessment in patients with decompensated liver cirrhosis: Implications for peritoneal dialysis

**DOI:** 10.1038/s41598-020-59817-3

**Published:** 2020-02-18

**Authors:** Elisabeth Schwaiger, Amrei Simon, Peter Wabel, Benjamin Schairer, Carolin Berner, Lorenzo Signorini, Matthäus Ernstbrunner, Rayko Evstatiev, Philipp Schwabl, Georg Hinterholzer, Florian Frommlet, Andreas Vychytil, Christian J. Müller, Manfred Hecking

**Affiliations:** 1 Department of Internal Medicine III, Clinical Division of Nephrology and Dialysis, Medical University of Vienna, Vienna, Austria; 2 Department of Internal Medicine II, Kepler University Hospital, Med Campus III, Linz, Austria; 3 Fresenius Medical Care, Research and Development, Bad Homburg, Germany; 4 1st Medical Department, Division for Nephrology, Sozialmedizinisches Zentrum Süd, Vienna, Austria; 5 Dipartimento di Nefrologia e Dialis, Azienda Ospedaliera Universitaria Integrata, Verona, Italy; 6 Department of Anaesthesiology and Critical Care Medicine, Medical University of Vienna, Vienna, Austria; 7 Department of Internal Medicine III, Clinical Division of Gastroenterology and Hepatology, Medical University of Vienna, Vienna, Austria; 8 Center for Medical Statistics, Informatics and Intelligent Systems, Medical University of Vienna, Vienna, Austria

**Keywords:** Gastroenterology, Outcomes research, Peritoneal dialysis

## Abstract

Bioimpedance spectroscopy (BIS) is routinely used in peritoneal dialysis patients and might aid fluid status assessment in patients with liver cirrhosis, but the effect of ascites volume removal on BIS-readings is unknown. Here we determined changes in BIS-derived parameters and clinical signs of fluid overload from before to after abdominal paracentesis. Per our pre-specified sample size calculation, we studied 31 cirrhotic patients, analyzing demographics, labs and clinical parameters along with BIS results. Mean volume of the abdominal paracentesis was 7.8 ± 2.6 L. From pre-to post-paracentesis, extracellular volume (ECV) decreased (20.2 ± 5.2 L to 19.0 ± 4.8 L), total body volume decreased (39.8 ± 9.8 L to 37.8 ± 8.5 L) and adipose tissue mass decreased (38.4 ± 16.0 kg to 29.9 ± 12.9 kg; all *p* < *0.002*). Correlation of BIS-derived parameters from pre to post-paracentesis ranged from R² = 0.26 for body cell mass to R² = 0.99 for ECV. Edema did not correlate with BIS-derived fluid overload (FO ≥ 15% ECV), which occurred in 16 patients (51.6%). In conclusion, BIS-derived information on fluid status did not coincide with clinical judgement. The changes in adipose tissue mass support the BIS-model assumption that fluid in the peritoneal cavity is not detectable, suggesting that ascites (or peritoneal dialysis fluid) mass should be subtracted from adipose tissue if BIS is used in patients with a full peritoneal cavity.

## Introduction

Liver cirrhosis impairs both the splanchnic and the systemic circulation resulting in a “hyperdynamic circulatory syndrome”^1–3^. Portal hypertension causes vasodilation in the splanchnic circulation, and thereby ascites and hepatorenal syndrome^1,4^. Adaption mechanisms to portal hypertension in the systemic circulation can lead to decreased systemic vascular resistance, decreased arterial blood pressure and increased cardiac output and heart rate, potentially triggering cardiomyopathy^1,2,4–6^. Optimizing fluid volume therefore is crucial in treating patients with liver cirrhosis.

The clinical evaluation of the fluid status of patients with liver cirrhosis is challenging. Intravascular volume depletion can coexist with edema and ascites, so that the application of diuretics aimed at diminishing edema or ascites can lead to additional intravascular volume depletion and kidney injury. Application of parenteral fluid can potentially worsen ascites, pleural effusion or heart failure^7–9^.

Technical devices which employ bioimpedance spectroscopy (BIS) to evaluate fluid status and tissue composition can help assessing the fluid status in patients with decompensated liver cirrhosis^7^. In nephrology, the benefit of using the Body Composition Monitor (BCM) to evaluate ‘normohydration weight’^10,11^ has been recognized for hemodialysis patients^12–14^, as well as for peritoneal dialysis (PD) patients^15^. Prospective studies aiming at improving hard outcomes in PD patients using BCM-BIS are ongoing^16,17^ and have recently been completed^18^.

Filling of the peritoneal cavity occurs both in cirrhotic patients with portal hypertension, who develop ascites^19,20^, and in patients undergoing PD. Both patient groups may undergo large and relatively quick fluctuations of the ‘third space’. Possible effects of the filling of the peritoneal cavity on BIS results were subject of previous discussions^21–23^. The disunity is mirrored by different filling status throughout various studies using BIS: some researchers measured with a full abdomen^24–26^, others after the abdomen was drained^27–30^, or did not elaborate on the matter, assuming there is no difference^31,32^.

In the present study we performed BCM-BIS measurements in patients with decompensated liver cirrhosis before and after ascites paracentesis in order to (1) evaluate the fluid status of patients with decompensated liver cirrhosis, (2) compare BCM-BIS results with the clinical assessment of fluid overload (FO) and (3) examine the effect of peritoneal filling on the BCM-BIS results. Our aim was not only to further evaluate BCM-BIS in patients outside of the nephrological scenario, but also to use the clinical setting of removing large ascites volumes in liver cirrhosis patients as a model for PD patients, where the influence of peritoneal cavity filling on BIS results is currently still unclear.

## Results

### Characteristics of the study population

Twenty-five of 31 patients were males and mean age of the cohort was 59.5 ± 11.2 years (for details of the patient cohort see Table 1). The etiology of the liver cirrhosis causing ascites was as follows: 14 patients suffered from alcohol-induced liver cirrhosis, 7 patients from viral hepatitis, 7 patients from liver cirrhosis of unknown origin, 1 patient had autoimmune hepatitis causing liver cirrhosis, 1 patient had Budd-Chiari syndrome and 1 patient had non-alcoholic steatohepatitis. All patients had ascites and accordingly, all patients had Child-Pugh Score > A (20 patients with Child-Pugh Score B, 11 patients with Child-Pugh Score C, Table 1).

**Table 1:** Demographic data and clinical characteristics of the whole study population.

	All	Normal range
Number of patients, N (%)	31 (100.0)	
Age [years], mean ± SD	59.5 ± 11.2	
Male sex, N (%)	25 (80.6)	
Height [cm], mean ± SD	175.4 ± 11.2	
Weight [kg], mean ± SD	79.9 ± 16.2	
Body mass index [kg/m²], mean ± SD	26.0 ± 4.8	
“Dry weight” [kg], mean ± SD	76.6 ± 15.4	
Child-Pugh Score B, N (%)	20 (64.5)	
Child-Pugh Score C, N (%)	11 (35.5)	
Number of patients with diuretics	20 (64.5)	
Serum albumin [g/L], mean ± SD	30.3 ± 5.5	35–52
Alanine aminotransferase [U/L], median (IQR)	29 (14–54)	12–46
Aspartate aminotransferase [U/L], median (IQR)	59 (29–118)	10–55
Alkaline phosphatase [U/L], median (IQR)	149 (98–362) (male) 70 (57–126) (female)	40–130 (male) 35–105 (female)
Gamma-glutamyltransferase [U/L], median (IQR)	159 (80–436) (male) 26 (14–82) (female)	60 (male) 40 (female)
Bilirubin [mg/dL], median (IQR)	1.5 (0.9–3.9)	0.3–1
International Normalized Ratio, mean ± SD	1.5 ± 0.3	
Serum creatinine [mg/dL], mean ± SD	1.5 ± 1.0 (male) 0.8±0.4 (female)	0.70–1.20 (male) 0.50–0.90 (female)
Blood urea nitrogen [mg/dL], median (IQR)	19.5 (10.5–58.5)	8–23
Blood urea nitrogen/serum creatinine, median (IQR)	17.5 (11.7–32.2)	
Serum sodium [mEq/L], mean ± SD	134.0 ± 6.2	136–145
Serum potassium [mmol/L], mean ± SD	4.3 ± 0.8	3.5–5.1
Systolic blood pressure [mmHg], mean ± SD	113 ± 14	90–140
Diastolic blood pressure [mmHg], mean ± SD	68 ± 9	60–80
Heart rate [min^−1^], mean ± SD	86 ± 12	60–100
Edema, N (%)	20 (64.5)	
Volume of abdominal paracentesis [L], mean ± SD	7.8 ± 2.6	
Duration of abdominal paracentesis [min], mean ± SD	126.2 ± 39.3	
Patients receiving human albumin, N (%)	30 (96.7)	
Substituted human albumin (g), median (IQR)	57.6 (38.4–76.8)	

All continuous parameters are presented as means ± standard deviations.

Mean serum creatinine of the study cohort was elevated above the normal range (shown in Table 1) in 13 men whose mean creatinine was 2.2 ± 1.0 mg/dL and in 2 women whose mean creatinine was 1.2 ± 0.4 mg/dL. Nineteen patients had hyponatremia below 136 mEq/L, but were neurologically asymptomatic; their mean serum sodium levels were 130.2 ± 4.9 mEq/L. Eighteen patients had normal heart rate and blood pressure (normal ranges shown in Table 1). Twenty patients had edema. Twenty patients were using diuretics at the time of the measurement. Twenty-three patients presented with serum albumin values below the normal range, their mean serum albumin was 27.9 ± 3.8 g/L. Mean volume of the abdominal paracentesis was 7.8 ± 2.6 L, mean duration of the procedure was 126.2 ± 39.3 minutes. Thirty patients received human albumin, the median human albumin substitution amounted to 57.6 g (IQR: 38.4–76.8 g).

### BCM-BIS derived fluid status before abdominal paracentesis

Mean TBV in 26 patients was 39.9 ± 9.8 L, mean ECV was 20.2 ± 5.2 L, mean ICV was 19.7 ± 5.1 L and mean absolute FO was 3.5 ± 2.6 L (Table 2). Sixteen of 31 patients (51.6%) had FO ≥ 15% ECV, thereby classifying as being fluid overloaded. In the entire patient cohort, mean relative FO was 16.1 ± 10.4%. In the fluid overloaded subgroup, mean relative FO was 23.3 ± 5.2% and mean absolute FO was 4.8 ± 1.9 L.

**Table 2:** Results of the Body Composition Monitor (BCM)-measurements before and after abdominal paracentesis (N = 26)*.

	Results before paracentesis	Results after paracentesis	Mean patient difference	p-value
Fluid Overload (FO) [L]	3.5 ± 2.6 L	3.5 ± 2.6 L	0.0 ± 1.6 L	0.89
Relative FO [% of ECV]	16.1 ± 10.4%	17.6 ± 11.7%	1.5 ± 7.8%	0.34
TBV [L]	39.9 ± 9.8 L	37.8 ± 8.5 L	−2.1 ± 3.1 L	0.002
ECV [L]	20.2 ± 5.2 L	19.0 ± 4.8 L	−1.2 ± 0.7 L	<0.001
ICV [L]	19.7 ± 5.1 L	18.8 ± 4.3 L	−0.9 ± 3 L	0.14
ECV/ICV	1.0 ± 0.2	1.0 ± 0.2	−0.02 ± 0.2	0.55
Adipose tissue mass [kg]	38.4 ± 16.0 kg	29.9 ± 12.9 kg	−8.4 ± 10.2 kg	<0.001
Lean tissue mass [kg]	39.4 ± 12.6 kg	38.7 ± 10.2 kg	0.7 ± 8.4 kg	0.66
Lean tissue mass percentage	49.01 ± 12.6%	54.0 ± 11.9%	4.9 ± 10.4%	0.024
Fat mass [kg]	27.4 ± 10.5 kg	22.0 ± 9.5 kg	−5.4 ± 5.4 kg	<0.001
Fat mass percentage	33.8 ± 9.8%	29.7 ± 9%	−4.0 ± 6.4%	0.004
Fat Tissue Index[kg/m^2^]	12.1 ± 4.8 kg/m^2^	9.7 ± 4.2 kg/m^2^	−2.6 ± 2.4 kg/m^2^	<0.001
Body cell mass [kg]	21.0 ± 8.5 kg	20.5 ± 6.7 kg	−0.5 ± 6.0 kg	0.66

All variables in the second and third vertical column are means ± standard deviations. Values in the fourth column were derived by averaging the results of the formula: BCM-BIS result after abdominal paracentesis minus BCM-BIS result before abdominal paracentesis.

Abbreviations: TBV = total body volume, ECV = extracellular volume, ICV = intracellular volume, ECV/ICV: Quotient of extracellular volume and intracellular volume, NH = normally hydrated.

*As explained in the Methods under Study details.

### Correlation between BCM-BIS derived FO and clinical symptoms

The clinical fluid status of the study patients, evaluated before the BCM-BIS measurement, did not correlate with the presence of BCM-BIS derived FO prior to the abdominal paracentesis (Table 3). Specifically, among 20 patients who had edema, 12 patients had BCM-BIS derived FO ≥ 15% ECV (*p* = *0.27*, Table 3). This result remained similar after the abdominal paracentesis, where among 20 patients who were classified as having edema, 15 patients had BCM-BIS derived FO ≥ 15% ECV (*p* = *0.42*). Similarly, we did not observe any correlation between BCM-BIS derived FO and the treatment with diuretics, either before or after the abdominal paracentesis (*p* = *1.0* and *p* = *1.0*, respectively). Finally, we did not observe a significant correlation between the use of diuretics and edema (*p* = *1.0*).

**Table 3:** Correlation between clinical findings and BCM-BIS results.

Before paracentesis			
*p* = *0.273*	Fluid overload <15% ECV → NO	Fluid overload ≥ 15% ECV → YES	Total
Edema → NO	7	4	11
Edema → YES	8	12	20
Total	15	16	31
***p*** **=** ***1.0***	**Fluid overload <15% ECV → NO**	**Fluid overload ≥ 15% ECV → YES**	**Total**
Diuretics → NO	5	6	11
Diuretics → YES	10	10	20
Total	15	16	31
**After paracentesis**			
***p*** **=** ***0.423***	**Fluid overload <15% ECV → NO**	**Fluid overload ≥ 15% ECV → YES**	**Total**
Edema → NO	5	6	11
Edema → YES	5	15	20
Total	10	21	31
***p*** **=** ***1.0***	**Fluid overload <15% ECV → NO**	**Fluid overload ≥ 15% ECV → YES**	**Total**
Diuretics → NO	3	8	11
Diuretics → YES	7	13	20
Total	10	21	31

ECV = extracellular volume; Fluid overload was defined as fluid overload/ECV*100 ≥15%. Diuretic therapy was defined as having at least 1 diuretic drug at the time of measurement.

### BCM-BIS derived fluid status and body composition before and after abdominal paracentesis

**Figure 1:**
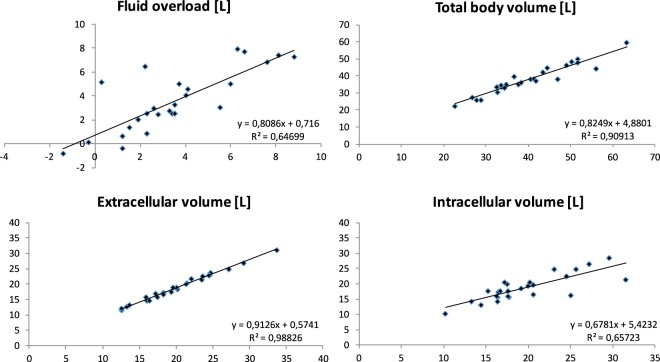
Scatterplot of different body fluid volumes. X-coordinate: full peritoneal cavity, y-coordinate: empty peritoneal cavity (N = 26)*. *As explained in the Methods under Study details.

**Figure 2:**
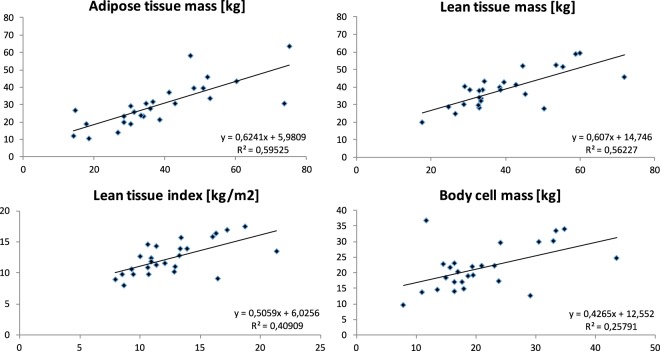
Scatterplot of different body tissue compartments. X-coordinate: full peritoneal cavity, y-coordinate: empty peritoneal cavity (N = 26)*. *As explained in the Methods under Study details.

The correlations of BCM-BIS derived fluid parameters from before to after abdominal paracentesis were very high for the total body volume (R² = 0.91) and the extracellular volume (R² = 0.99, Fig. 1). The biggest change in the BCM-BIS derived parameters was observed for adipose tissue mass (ATM). ATM changed by −8.4 ± 10.2 kg reflecting more than the complete volume change caused by the paracentesis of 7.8 ± 2.6 L. Further, significant differences were observed for the fat mass (−5.4 ± 5.4 kg), the ECV (−1.2 ± 0.7 L) and the TBV (−2.1 ± 3.1 L). FO, relative FO, LTM and ICV did not change significantly after paracentesis (Table 2). The correlation of BCM-BIS derived parameters for ATM was R² = 0.59 and for LTM was R² = 0.41 (Fig. 2).

## Discussion

The present study shows that (i) more than half (51.6%) of our study cohort patients were fluid overloaded, (ii) the results of the clinical assessment of FO differed significantly from the results of the BCM-BIS device and (iii) the removal of ascites did not influence the FO or LTM results obtained by BIS using the BCM-BIS device. The study results indicate that the working hypothesis of the third spaces holds true: The ascites in the peritoneal cavity was not detected by BIS, because even large changes in the ascites volume had no effect on FO, relative FO or LTM. This finding is important, as it shows the independence of BCM-BIS derived fluid status assessment from the ascites stage.

In patients with liver cirrhosis, evaluation of the fluid status is essential for clinical decision making. Patients with liver cirrhosis develop a hyperdynamic circulation which in the course of the disease causes central arterial and vascular underfilling^33–35^ as well as FO^36^: The splanchnic blood volume is increased, accounting for more than 22% of the circulating volume^34^, combined with up to 50% of the ECV in the extravascular space^7^. Thus, the volume status of a cirrhotic patient is difficult to evaluate, and clinical signs of FO in this patient cohort can be misleading. BCM-BIS derived parameters could potentially provide therapeutically useful information on volume status. For instance, diuretics in patients with liver cirrhosis must be used cautiously, since both over- and underdosing have serious side effects^36,37^. BCM-BIS is limited in its inability to distinguish between interstitial and intravascular extracellular volume. Patients in the decompensated state, however, have an enlarged total blood volume^38–40^. Further studies should therefore evaluate the relationship between total blood volume and BIS-based assessment of fluid status.

In nephrology, where BCM-BIS is an established tool to evaluate the fluid status of hemodialysis and PD patients, ECV as well as FO are of primary clinical importance. The fact that both FO and relative FO were not affected by the paracentesis is also very important for the use of BCM-BIS in peritoneal dialysis patients. However, withdrawal of a large ascites volume causes pressure dynamics to change, which, in turn might lead to fluid flowing back into the peritoneal cavity^41^, thereby perhaps removing BCM-BIS detectable fluid from the extracellular space. Although ECV changed significantly, the mean patient difference of ECV with peritoneal filling and ECV after abdominal paracentesis was only slightly above our threshold of 1.0 L (1.2 L). The ECV change might also have been a consequence of the temporally overlapping albumin substitution enhancing the colloid osmotic pressure and thereby causing an intravascular fluid shift^42^.

For researchers who are unfamiliar with the BCM-BIS model, the reason why adipose tissue mass changed after paracentesis may be unclear. The measurement principle of bioimpedance relies on an alternating current being applied to the body, and measuring the impedance response. Volumes that are surrounded by a tissue wall (pericardium, bladder, peritoneum), however, cannot be penetrated by the current. The ascites volume, although contained in the weight of the patients, is not reflected in the bioimpedance-derived volume assessment. In the BCM-BIS model, adipose tissue is the residuum after the calculation of the lean tissue mass and fluid overload. Fluctuations of the body weight which are not reflected in the measurement of the bioimpedance are therefore reflected in the adipose tissue.

Only few other studies have evaluated the effect of peritoneal filling on ECV values measured by the BCM-BIS device, and the findings of these studies are somewhat diverse: Arroyo *et al*. found a significant increase in ECV after filling of 2 L into the peritoneal cavity^21^. Sipahi *et al*. demonstrated a higher conformity of BCM-BIS results with echocardiographic findings of left ventricular mass and left atrium volume in measurements with an empty peritoneal cavity, without any significant changes in BCM-BIS derived parameters^23^. Caron-Lienert *et al*. did not identify significant differences in any of the BCM-derived parameters and concluded that “BCM could be applied without the need for drainage of the dialysis fluid from the abdominal cavity of PD patients”^43^. Finally, Parmentier *et al*. did not register any significant changes in ECV, comparing BCM-BIS results of PD patients under PD treatment^22^.

A study by Keane *et al*.^44^ used various combinations of applying the BCM-BIS electrodes to PD patients (feet versus ankles) and thereby provides additional theoretical reasoning why the filling of the peritoneum only has limited impact on the determination of fluid overload using BCM-BIS. Nevertheless, fillings of the peritoneal cavity in the published studies were quite normal for PD patients (approximately 2 Liters). The BCM manual provided by Fresenius Medical Care states, that “PD patients can be measured irrespective of a filled or drained peritoneal cavity, and that the weight to be entered in the BCM - Body Composition Monitor is the patient’s weight with empty peritoneal cavity.” These instructions from the BCM manual are in accordance with the findings by Caron-Lienert *et al*.^43^.

One of the aims of the present study was to assess the impact of *large* quantities of fluid in the peritoneal cavity on the assessment of body composition and fluid overload. From pre- to post-paracentesis, we noted significant changes in ECV, and especially LTM. As the weight of the ascites volume is unknown before its removal, BCM-BIS measurements in patients with ascites should be performed with an empty abdomen, if clinicians have this choice. The BCM manufacturer on its website previously recommended measuring even PD patients with an empty abdomen, but this information was no longer accessible at the time of the manuscript submission. This previous website recommendation from Fresenius Medical Care, however, is somewhat in discrepancy with the instructions from the BCM manual. Whether the present study findings from liver cirrhosis patients can be extrapolated to PD patients is unclear, and the most conservative interpretation of our study findings may therefore be, that the BCM-BIS measurement procedure should always be standardized, as has been suggested in the “BCM application notes” from the years 2008. In agreement with Caron-Lienert *et al*.^43^, the present findings imply, if anything, that the patient weight should be entered correctly into the BCM-BIS device, and that this weight should exclude the mass of any intraabdominal fluid.

In patients with liver cirrhosis, malnutrition causing muscle wasting, sarcopenia^45^, and a decrease in fat mass, are very common^46–50^ and are linked with an increased mortality risk^46,48,49,51–54^. Despite its clinical importance, however, malnutrition is underdiagnosed, as conditions such as FO aggravate assessing the body composition in general and sarcopenia or a reduction of fat mass in particular^55,56^. Single-frequency and multi-frequency impedance have been shown to be adequate techniques for assessing the nutritional status of patients with liver cirrhosis^7,45,57–60^, with conflicting data on patients with cirrhosis and ascites in outdated studies^45,60–62^. The capacity of the BCM-BIS device to determine FO and to evaluate LTM and ATM could render it a helpful tool in the nutritional assessment. In our study, LTM was unaffected of peritoneal filling, so that in practice it might be easier to assess sarcopenia, which is associated with increased mortality in patients with liver cirrhosis^63,64^. Further studies are needed to determine the use of the practical use of the BCM in liver cirrhosis.

Edema and FO did not correlate in the present study, when evaluated by Fisher’s exact test. Moreover, we did not observe any correlation between BCM-BIS derived FO and the treatment with diuretics, either before or after the abdominal paracentesis (*p* = *1.0* and *p* = *1.0*, respectively), or between the use of diuretics and edema (*p* = *1.0*). In the nephrological community, BCM-BIS revealed the interesting phenomenon that a large proportion of hemodialysis patients (approximately 25% of patients with end stage kidney failure on maintenance hemodialysis or PD^65–68^) were fluid overloaded >15% ECV predialysis, despite the fact that these patients were under constant care of their nephrologist and dialysis care takers. Clinical experience shows that fluid overload is hard to detect^69^. To the best of our knowledge, there are no previous data on BCM-BIS and edema, but a previous study showed no correlation between pedal edema and inferior vena cava diameter, blood volume monitoring, plasma volume markers (all summarized as markers reflecting volume) in hemodialysis patients^70^. The challenge of adequately diagnosing fluid, and even more so of adequately treating FO through diuretics, is one of the central reasons for the potential value of introducing bioimpedance as well as other objective determinants of volume status into routine clinical practice^71,72^. Future studies are necessary to determine whether BCM-BIS guided fluid management by diuretics (not necessarily in patients on kidney replacement therapy, where such studies are already on the horizon) may improve outcomes.

Among this study’s limitations, we acknowledge that we did not include a comparator group, For nephrology (PD), the interpretation of our study conclusions is complex, as PD patients do not usually have vasodilation in the splanchnic circulation, the volume of the PD fluid is typically lower than ascites, and the intention of PD dialysate is (in many cases) to pull fluid from the extracellular space (ECV). However, our results are overall confirmative of the technical suggestions provided by the manufacturer, that PD patients should be measured with an empty cavity. One of the highlights of our study is the disparity between clinical assessment of FO and BCM-BIS derived FO, but the limitation is that the applicability of the BCM-BIS model to patients with liver cirrhosis is speculative. The present study was neither designed, nor powered to assess hard clinical outcomes. Another concern might be the study’s sample size calculation, based on previous findings from a cohort of female patients undergoing gynecological surgery, who were otherwise healthy^73^. This sample size calculation was based on a standard deviation of 2 L for ECV, as noted in the previous study, but the actual standard deviation for ECV before paracentesis in cirrhotic patients, identified in the present study was much higher (5.2 L). Formally speaking, our previous sample size calculation is therefore not informative of the primary endpoint, and the sample size should have been increased. The primary endpoint (difference of measured ECV in patients from before to after abdominal paracentesis), however, was significant, despite the study’s smaller sample size. Further study limitations include the fact that, although we recorded hemodynamic variables (blood pressure and heart rate) at baseline, our study is missing more sophisticated information, such as from echocardiographic assessment, and we also did not evaluate possible hemodynamic changes from pre- to post-paracentesis, in relationship to FO. Finally, we were unable to provide the protein and albumin concentration in the drained ascites fluid, which would have been useful to document that the ascetic fluid in all patients was transudate with a correspondingly low serum ascites albumin gradient^74^.

However, our study was designed to make use of the large volume shifts during abdominal paracentesis, in order to determine whether an immense filling of the peritoneal cavity with osmotically largely ineffective volume might influence the BCM-BIS results. We found that the fat measurements differed greatly from pre- to post-abdominal paracentesis suggesting that ‘missing weight’ from the ascites was attributed to the fat compartment. These observations lead to the simple conclusion that the mass of ascites (or peritoneal dialysis fluid) volume should be subtracted from a patient’s body weight, if BCM-BIS is used in patients with a full peritoneal cavity. Ultimately, our study also suggests that frequent evaluation of the fluid status using (BCM-) BIS may provide useful information for the treatment and prognosis of liver cirrhosis patients, in addition to clinical judgement, but further analyses with adequate follow-up are necessary to determine whether such endeavors might translate into improved outcomes.

## Methods

### Body composition monitor

The BCM uses a non-invasive technique^75^. Here, we conducted all BCM-BIS measurements in accordance with the manufacturer’s instruction (Fresenius Medical Care, Bad Homburg, Germany): After the patient had rested for at least 5 minutes in supine position, 2 non-recyclable electrodes were affixed to wrist and ankle, respectively. The BCM electrodes were connected with a cable, provided by the manufacturer. Basic demographic data of the patient (sex, age, height, weight) were entered into the BCM-BIS device.

The BCM-BIS device generates resistance and reactance values at 50 distinctive frequencies in the range of 5 to 1000 kHz. The mathematical algorithm is based on physiologic tissue properties of normohydrated lean and adipose tissue^76^. The BCM determines extracellular (ECV), intracellular (ICV) and total body volume (TBV)^77^, as well as lean tissue mass (LTM), adipose tissue mass (ATM) and fat mass (FM). [Adipose tissue, per definition of body composition experts, contains the lipid inside the fat cell, the small amount of intracellular water in the fat cell, and the extracellular water in the normohydrated adipose tissue. Fat, however, is lipid mass only, without the intracellular or extracellular water^76^]. The difference between measured and physiological ECV is defined as the fluid excess (= fluid overload, in liters (FO) and in % of ECV^76^. The underlying assumption postulates that fluid in third spaces (e.g. peritoneal cavity, bladder, gut) will not be penetrated by the electric current used for the BCM-BIS device; therefore, the filling of these spaces remain invisible for the assessment. Potential volume in these spaces will be regarded as adipose tissue mass (ATM) by the device, and not as FO.

A general note: In accordance with other authors^73^, we did not adopt the nomenclature included in the BCM-BIS device (i.e. of ‘overhydration’ and ‘extracellular water’). Although overhydration, FO and volume overload are often used synonymously, ‘hydration’ strictly refers to water whereas ‘volume expansion’ describes the accumulation of isotonic fluid (salt and water). We therefore replaced the term overhydration by ‘FO’ and coherently replaced the terms extracellular water, total body water and intracellular water by ECV, TBV and ICV (using volume [V] instead of water).

### Study details

BCM-BIS measurements were implemented into routine clinical practice of the Department of Gastroenterology and Hepatology on 1-June-2016. From that day onward until 1-July-2017, patients with ascites caused by liver cirrhosis, identified through the ward and the outpatient clinic, were measured using the BCM, and were carefully examined. BCM-BIS measurements were undertaken before and after abdominal paracentesis by 2 independent investigators. In case of an insufficient quality of the measurements (quality of data below 70%), the measurement was repeated. N = 5 BCM-BIS results contained missing data for several body composition parameters and were discarded.

The actual body weight is an essential input for the algorithm used in the BCM. The body weight of the second measurement (after paracentesis) was obtained by subtracting the ascites volume from the initial weight under the presupposition that 1 L of withdrawn ascites volume equals 1 kg of body weight. Clinical signs of FO (peripheral edema) were evaluated before the first measurement, and so were heart rate and blood pressure. Edemas were assessed by pressing the index finder of the dominant hand with moderate pressure into each lower extremity of the patient at the pretibial region, recording the clear appearance of an indentation as “edema”. In accordance with a previous study^70^, the extent of the edema appearance was not graded.

Demographic data of all patients included age, height, body weight, body mass index and etiology of liver cirrhosis. We also recorded whether a patient was treated with diuretic agents. To assess kidney and liver function, we obtained the following laboratory parameters: serum albumin, alanine aminotransferase, aspartate aminotransferase, alkaline phosphatase, gamma-glutamyl transferase, bilirubin, international normalized ratio, serum creatinine, blood urea nitrogen, blood urea nitrogen/serum creatinine, serum potassium and serum sodium concentration. Child-Pugh Score was calculated on the basis of serum albumin, bilirubin, international normalized ratio, sonographic evaluation of ascites and symptoms of hepatic encephalopathy.

We registered the time between BCM-BIS measurements, the volume of the abdominal paracentesis, all BCM-BIS derived values and the volume of the albumin infusion administered after abdominal paracentesis. Patients received 8 g of human albumin per liter of ascites fluid removed, using a 20% formulation (manufactured by CSL Behring GmbH) containing at least 19.2 g albumin per 100 mL infusion solution The second BCM-BIS measurement followed the abdominal paracentesis including the albumin substitution but did not exclude a temporal overlap.

To evaluate the obtained data in the form of the present open, non-interventional, observational cohort study, we obtained approval from the Ethics Committee of the Medical University of Vienna (EK No.: 2096/2016) and anonymized all participant information. All study details were therefore obtained retrospectively from analyses performed per routine clinical practice. The Ethics Committee approved the study despite the fact that patients had not provided written informed consent, acknowledging the fact that obtaining written informed consent would not have been possible retrospectively from some of the patients who had died only a few weeks after they had received their ascites puncture. The study adhered to the Declaration of Helsinki.

### Sample size calculation and statistical methods

The primary endpoint was defined, per our pre-specified study protocol, as the difference (delta) of measured ECV in patients from before to after abdominal paracentesis. Secondary endpoints included the deltas of measured TBV and ICV in patients from before to after abdominal paracentesis and also included the correlation between clinical signs of FO and a BCM-BIS derived classification of FO (%FO > 15% ECV).

The pre-specified sample size calculation was based on Student’s t-test for dependent samples. In our previous study^73^ the standard deviation of the ECV values in patients receiving perioperative fluid therapy was approximately 2 L. Using this standard deviation and assuming a moderate and thereby clinically meaningful within-subject correlation (r) of 0.5 and a beta value of 0.2, we arrived at the conclusion that 31 subjects would be needed to determine an effect size of 1 L (delta ECV from before to after abdominal paracentesis) at a statistical significance level of 0.05.

Descriptive statistics (mean and standard deviation for normally distributed variables, median and interquartile range for not normally distributed variables) were employed to depict patients’ characteristics, laboratory values and BCM-BIS derived results. All deltas of the BCM-BIS measurement were evaluated using the 2-sided, paired Student’s t-test and Pearson’s correlation analysis.

A patient was considered to be fluid overloaded, when relative FO was equal to or exceeded 15% ECV. The evaluation of the fluid status was obtained before paracentesis (in patients with a full abdomen). We used Fisher’s exact test to analyze the association between clinical signs of FO (classified by the presence of peripheral edema) and BCM-BIS derived FO, and between diuretic therapy and BCM-BIS derived FO.

For calculations we used MS Excel 2007 (Microsoft corporation®, Redmond, Washington, USA) and IBM SPSS Statistics 25.0. (IBM SPSS 25.0, IBM corporation®, Armonk, NY, USA). P-values < 0.05 were considered statistically significant.

## Data Availability

Data supporting the results are available upon request.
